# Lesion Geometry as Assessed by Optical Coherence Tomography Is Related to Myocardial Ischemia as Determined by Cardiac Magnetic Resonance Imaging

**DOI:** 10.3390/jcm10153342

**Published:** 2021-07-28

**Authors:** Rosalia Dettori, Andrea Milzi, Michael Frick, Kathrin Burgmaier, Mohammad Almalla, Richard Karl Lubberich, Nikolaus Marx, Sebastian Reith, Mathias Burgmaier

**Affiliations:** 1 Department of Internal Medicine I, University Hospital of the RWTH Aachen, 52070 Aachen, Germany; rdettori@ukaachen.de (R.D.); mfrick@ukaachen.de (M.F.); malmalla@ukaachen.de (M.A.); rlubberich@ukaachen.de (R.K.L.); nmarx@ukaachen.de (N.M.); sreith@ukaachen.de (S.R.); mburgmaier@ukaachen.de (M.B.); 2 Department of Pediatrics, University Hospital of Cologne, 50937 Cologne, Germany; kathrin.burgmaier@uk-koeln.de

**Keywords:** optical coherence tomography, cardiac magnetic resonance imaging, coronary artery disease, stenosis geometry

## Abstract

Introduction: Although the relationship between the geometry of coronary stenosis and the presence of myocardial ischemia is well known, the association between stenosis geometry and severity and/or extent of ischemia is still unexplored. Thus, we investigated this relationship using optical coherence tomography (OCT) to assess stenosis parameters and cardiac magnetic resonance imaging (CMR) to determine both extent and severity of ischemia. Methods: We analyzed 55 lesions from 51 patients with stable angina. Pre-interventionally, all patients underwent OCT-analysis of stenosis morphology as well as CMR to determine both the extent and severity of myocardial ischemia. Results: Percent area stenosis (%AS) was significantly associated with ischemic burden (r = 0.416, *p* = 0.003). Similar results could be obtained for other stenosis parameters as well as for several other parameters assessing the extent of ischemia. Furthermore, OCT-derived stenosis parameters were associated with the product of ischemic burden and severity of ischemia (%AS: r = 0.435, *p* = 0.002; similar results for other parameters). A Poiseuille’s-law-modelled combination of stenosis length and minimal lumen diameter yielded a good diagnostic efficiency (AUC 0.787) in predicting an ischemic burden >10%. Conclusions: Our data highlight the key role of the geometry of coronary lesions in determining myocardial ischemia.

## 1. Introduction

In chronic coronary syndromes (CCS), the stable form of coronary artery disease, revascularization of intermediate coronary stenoses is indicated in the presence of documented ischemia [1]. Such an approach yields a sustained clinical benefit for patients [2,3]. Various methods may be employed in order to assess the hemodynamic relevance of a lesion and, thus, the presence of ischemia. Among these, the gold standard is represented by a physiological assessment during invasive coronary angiographies, such as fractional flow ratio (FFR) or instantaneous wave-free ratio (iwFR). However, a valuable alternative is represented by non-invasive testing for ischemia, especially imaging-based tests as single positron emission coronary tomography and cardiovascular magnetic resonance imaging (CMR). CMR, in particular, has the advantage to be able to provide precise information about the ventricular function, myocardial perfusion, edema and viability in a single examination [4]; furthermore, a non-pathological stress CMR in patients with CCS is associated with a very small occurrence of adverse cardiac events [5,6], and CMR-based management of patients with CCS showed non-inferiority at 12 months compared to a more invasive, FFR-based management [7].

Aside from the sheer presence of ischemia, though, a central role is played by the extent of the ischemic myocardium. In fact, a larger area of ischemia is associated with a worse long-term prognosis in the absence of coronary intervention [8], so that an ischemic burden >10% is integrated into the decision tree for the interventional management of patients with CCS included in the current European guidelines [1]. Of course, the ability to assess such a prognostically relevant feature represents a significant advantage of image-based ischemia testing [4]. Previous studies consistently reported that the presence of ischemia can be predicted by lesion geometry [9,10,11,12,13,14,15,16,17], at least to a certain extent. However, it is still unexplored whether the geometric features of coronary stenosis are also able to predict the extent of myocardial ischemia, which as previously discussed allows a further stratification of the risk for future events without revascularization. Clarifying this relationship between the anatomic severity of coronary lesions and the extent of ischemia was the aim of the present study.

In order to obtain a precise assessment of the geometry of coronary stenoses, we employed optical coherence tomography (OCT), an intravascular imaging modality which, thanks to its supreme resolution, may be considered the gold standard in the evaluation of luminal and periluminal structures. On the other hand, the extent of ischemia was analyzed through CMR, which provides the highest resolution among the imaging modalities assessing ischemic myocardium.

## 2. Materials and Methods

### 2.1. Study Population

We retrospectively enrolled 51 patients with CCS (defined as lack of progression in the last 6 weeks) undergoing coronary angiography and stress perfusion CMR at the Department of Cardiology, University Hospital of the RWTH Aachen, Germany. Previous studies partly included patients in this study cohort [13,15,16,17,18,19,20,21,22,23,24,25,26,27]. Inclusion criteria were suspected CCS with or without evidence of ischemia in CMR and an at least intermediate-grade coronary stenosis (40–90%) suitable for OCT-analysis. Exclusion criteria were left main coronary artery stenosis, bypass graft lesions, culprit lesions of a former myocardial infarction, lesions in coronary vessels supplying non-viable myocardium, serial lesions within the same vessel, in-stent restenosis, acute coronary syndromes, hemodynamic or rhythmic instability, acute or chronic renal insufficiency (serum creatinine > 1.5 mg/dL) and coronary vessels with chronic total occlusion, severe tortuousness or calcification impeding the safe advancing of the OCT catheter. Informed consent of all patients was obtained prior to inclusion in the study. The study was approved by the local Ethics Committee (Independent Ethics Committee of the Medical Faculty of the RWTH Aachen) and is in accordance with the Declaration of Helsinki on ethical principles for medical research involving human subjects.

### 2.2. OCT Image Acquisition and Analysis

The OCT images in the target lesions were acquired using a Frequency-Domain-OCT C7XR system and the DragonFly catheter (St. Jude Medical Systems; Lightlab Imaging, Inc., Westford, MA, USA). Offline analysis was performed frame by frame in 0.2 mm intervals independently by two experienced observers using St. Jude proprietary software. A consensus measurement took place in case of divergent results. Before the coronary intervention, the following quantitative and qualitative measurements were taken: at the frame with the smallest intraluminal area, minimal lumen area (MLA) and minimal lumen diameter (MLD) were assessed. Reference lumen area was measured at the reference cross-section with the largest lumen within 10 mm proximal or distal to the MLA and before any side branch. Percent area stenosis (%AS) was calculated as ([reference lumen area − MLA]/reference lumen area) × 100. The segment around the MLA with a cross-sectional area of at least 50% in comparison to the reference segment lumen area corresponded to the stenosis length [13,15,24,28].

A calcified plaque was defined as a signal-poor region with defined borders, a fibrous plaque corresponded to a homogeneous, signal-rich region, while a lipid plaque was characterized by a signal-poor region with diffuse borders. A lipid-rich plaque with an overlying FCT ≤ 65 µm reaching at least two quadrants of the vessel circumference was defined as a TCFA. The minimal FCT was measured as the minimal distance between the arterial lumen and the inner border of the lipid pool, while the average over the entire length of the lipid plaque was defined as the mean FCT. Plaque instability was defined by a minimal FCT cut-off adjusted to 80 µm, according to previous studies [13,29].

As previously defined, macrophages were interpreted as “signal-rich, distinct, or confluent punctate regions that exceed the intensity of background speckle noise” [28]. Angular extension of macrophages corresponded to “macrophage arc” [22].

In lipid-rich plaque, measurement of lipid arc took place at every 1-mm interval throughout the complete length of each lesion and the average value was calculated. Measurement of the lipid length was performed on a longitudinal view. The lipid volume index was calculated by multiplying the averaged lipid arc by the lipid length [30,31].

### 2.3. CMR Image Acquisition and Analysis

CMR image acquisition took place before coronary intervention on a 1.5 Tesla magnetic resonance scanner (Achieva, Philips Healthcare, Best, The Netherlands). After standard cine imaging, contrast-enhanced first-pass perfusion imaging (3 short-axis slices per heartbeat, an intravenous bolus of Magnograf (Gadopentetat-Dimeglumin, 0.1 mmol/kg) followed by 30 mL of saline flush at 4 mL/second during vasodilator-stress with adenosine (140 µg/kg/min intravenously for 4 min)) was performed. Then, 10 min after the contrast injection (a second portion was given to add up to 0.2 mmol/kg), standard late gadolinium enhancement (LGE) imaging was executed.

CMR image analysis was conducted on a dedicated CMR workstation (ExtendedWorkspace, Philips Healthcare, Best, The Netherlands) by an experienced CMR cardiologist blinded to the results of the OCT examination.

**Figure 1:**
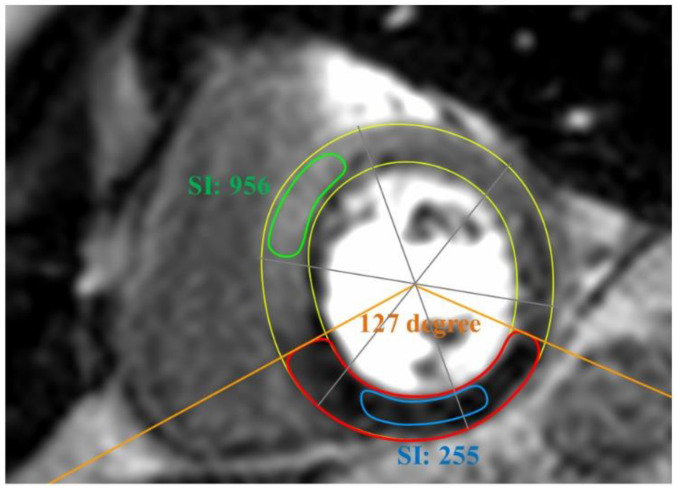
Exemplificative analysis of ischemia in CMR. Myocardial area: manual tracking of endocardial and epicardial border (yellow). Segmentation of the myocardium according to the standardized AHA 16 segment model (grey). Ischemic area (red) with signal intensity (SI ischemia) measured in the center (blue). Signal intensity (SI) of remote myocardium (green). The circumferential extent of ischemia (orange) is measured as the angle of ischemia with vertex at the center of the AHA 16 segment model. Ischemic signal intensity (SI) ratio: SI ischemia/SI remote × 100. Percentage of ischemic burden = ischemic area/total myocardial area × 100.

The first pass perfusion sequence was visually screened for the dynamic with the greatest extent of ischemia. The apical, mid-ventricular and basal short-axis (SA) slice of this dynamic was then used for further analysis. In the case of detection of several ischemic areas, single analyses were strictly separated according to the respective coronary artery supply territories. Concerning the LGE sequence, for each slice of perfusion, a corresponding SA-slice of LGE-imaging was used for scar quantification. Further analysis is demonstrated in Figure 1. For each SA-slice, the endocardial and epicardial borders were manually tracked to determine the myocardial area. The myocardium of each slice was divided according to the standardized AHA 16 segment model [32]. Then, for each slice, the region of ischemia was visually assessed. For determination of the effective ischemic myocardial area, an area of scar within an ischemic region was excluded. The percentage of ischemic burden was calculated as ischemic area divided by the total myocardial area × 100. Measurement of the angle of ischemia was performed by defining the center of the 16-segment AHA model as the vertex of the ischemic angle.

As a surrogate of the intensity of ischemia, we calculated the ischemic signal intensity (SI) ratio. To this aim, the SI of the darkest area (excluding a probable dark rim artifact at the endocardial border) within the ischemic area was designated. Further, the SI of a remote myocardial area (as visually assessed) was determined. The ischemic ratio was then calculated as SI ischemia/SI remote × 100. An exemplificative analysis of CMR images is shown in Figure 1.

### 2.4. Statistical Analysis

Categorical variables were summarized as count (percentage), continuous variables as mean ± standard deviation. Distributions of continuous variables were compared with *t*-test. The association of categorical variables was evaluated by Pearson’s Chi-squared test. In statistical testing, we did not account for multiple lesions in the same patient. In order to assess the association between OCT-derived intraluminal and intramural parameters with CMR-derived parameters assessing the extent and intensity of ischemia, we performed linear and non-linear regression analyses. To identify the optimal cut-off-value of various OCT-derived parameters in predicting an ischemic burden >10% of total myocardium [33,34] we performed receiver operating curve (ROC) analysis. Values with the highest Youden-index were identified as optimal cut-off values. A classification of the diagnostic efficiency according to the values of the area under the curve (AUC) was used as described elsewhere [35]. In order to evaluate the value of a combination of the significant OCT-derived parameters to predict an ischemic burden >10%, we performed multivariable logistic regression. ROC analysis was then performed based on the predictive values of this multiple regression model. All statistical analyses were performed with SPSS software (IBM Corp., Armonk, NY, USA). Statistical significance was awarded by *p* < 0.05.

## 3. Results

### 3.1. Clinical Characteristics

We analyzed a total of 55 lesions of at least intermediate severity (mean percent area stenosis: 75.0 ± 9.4%) from 51 patients with CCS. For patient and lesion characteristics please refer to Table 1 and Table 2.

### 3.2. Association between Coronary Lesion Characteristics and Ischemia

**Figure 2:**
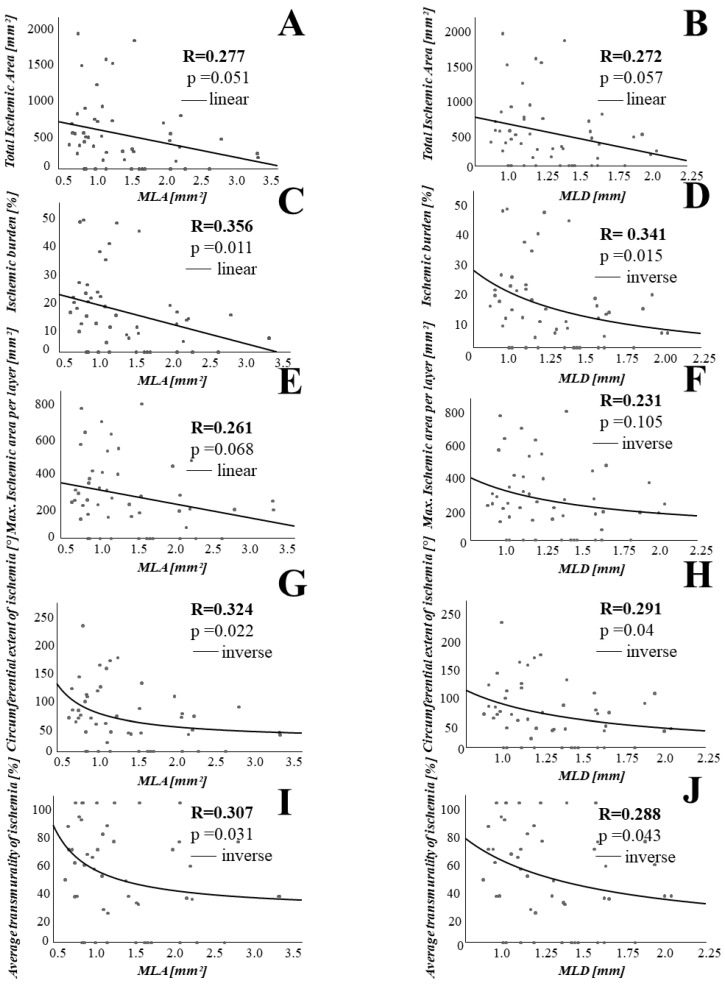
Minimal lumen area (MLA) and minimal lumen diameter (MLD) significantly correlate to extent of myocardial ischemia. On the X-axis, minimal lumen area (MLA) (**A**,**C**,**E**,**G**,**I**) and minimal lumen diameter (MLD) (**B**,**D**,**F**,**H**,**J**) are shown. On the Y-axis are plotted various parameters assessing the extent of myocardial ischemia.

**Figure 3:**
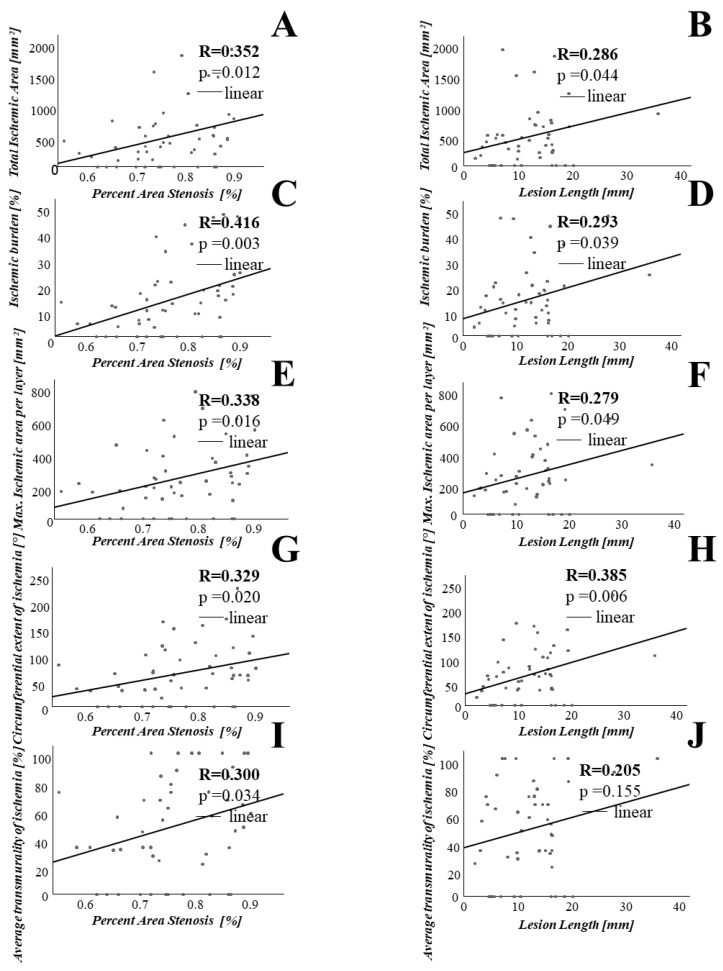
Percent area stenosis and lesion length significantly correlate with the extent of myocardial ischemia. On the X-axis, percent area stenosis (**A**,**C**,**E**,**G**,**I**) and lesion length (**B**,**D**,**F**,**H**,**J**) are shown. On the Y-axis are plotted various parameters assessing the extent of myocardial ischemia.

In order to investigate the association between lesion geometry as assessed by OCT and the extent of ischemia as measured with CMR, we performed linear and non-linear regression analyses. Percent area stenosis was significantly associated with various parameters assessing extent of ischemia, such as total ischemic area (r = 0.352, *p* = 0.012), total ischemic burden (r = 0.416, *p* = 0.003), maximal ischemic area per layer (r = 0.338, *p* = 0.016), circumferential extent of ischemia (r = 0.329, *p* = 0.020) and average transmurality of ischemia (r = 0.300, *p* = 0.034). Similar results could be obtained for other stenosis parameters, such as minimal lumen area (MLA) and minimal lumen diameter (MLD) and lesion length. Please refer to Figure 2 and Figure 3 for details.

**Figure 4:**
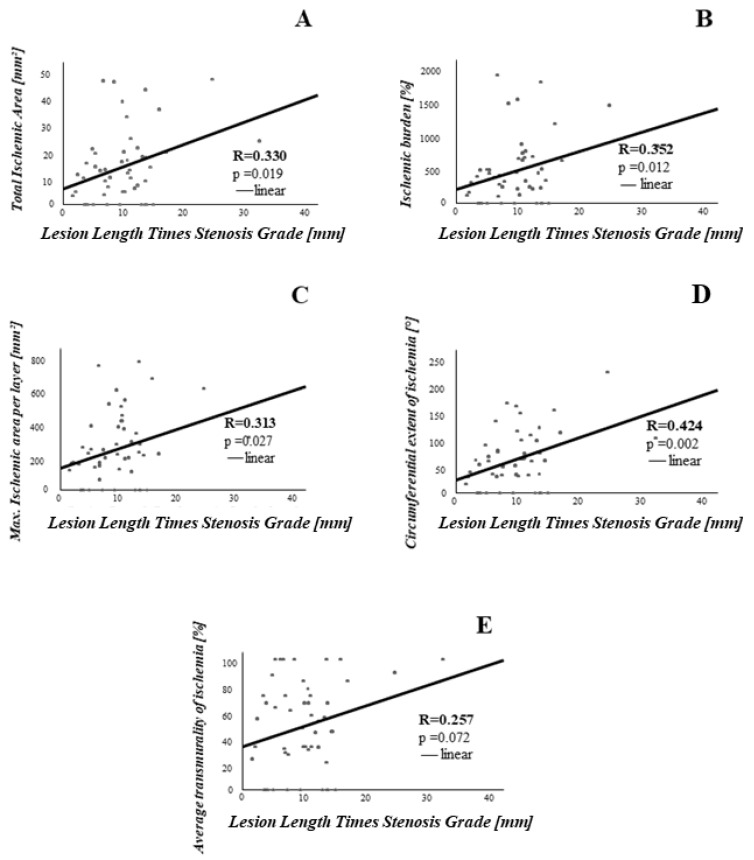
Lesion Length Times Stenosis Grade significantly correlates with extent of myocardial ischemia. On the X-axis, lesion length times stenosis grade (**A**–**E**) is shown. On the Y-axis are plotted various parameters assessing extent of myocardial ischemia.

To further analyze the association between the severity of coronary stenosis on the one hand and the extent of ischemia on the other hand, we defined lesion length times stenosis grade as the product of lesion length and percent area stenosis. Then, we assessed its association with the CMR parameters mentioned above. In this analysis, the product of lesion length and percent area stenosis was significantly associated with total ischemic area (r = 0.330, *p* = 0.019), ischemic burden (r = 0.352, *p* = 0.012), maximal ischemic area (r = 0.313, *p* = 0.027) and circumferential extent of ischemia (r = 0.424, *p* = 0.002). The results of these analyses are depicted in Figure 4.

Only proximal and medial coronary lesions were suitable for OCT-analysis. Still, in order to exclude lesion localization as a relevant predictor for the observed association between lesion geometry and ischemia, we performed regression analysis between the reference area, averaged between the non-diseased proximal and distal segments of the vessel, and ischemic burden. Here, no significant association could be found (r = 0.10, *p* = 0.807).

Since we observed a significant association between OCT-measured lesion characteristics and CMR-derived extent of ischemia, we next tested the association between stenosis parameters and severity of ischemia. The severity of ischemia in ischemic regions was assessed by comparing their signal intensity with other areas of the myocardium. In this analysis, we could detect a trend towards an association of MLA (r = 0.266, *p* = 0.066), MLD (r = 0.251, *p* = 0.086) and percent area stenosis (r = 0.285, *p* = 0.051) with SI ratio.

**Figure 5:**
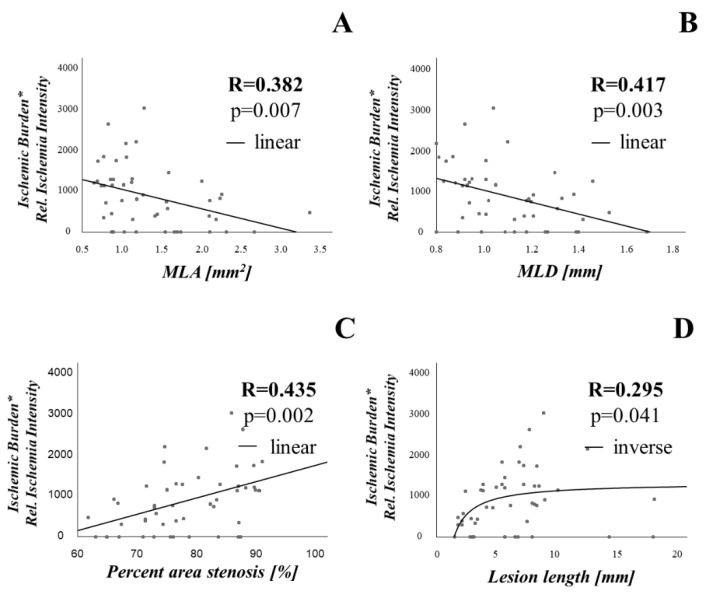
Stenosis geometry is associated with a composite index assessing both the extent and intensity of ischemia. As a surrogate parameter assessing both extent and intensity of ischemia, we calculated the product of ischemic burden and relative signal intensity of ischemic areas. This value, plotted on the Y-axis, was consistently associated with lesion geometry. The association with MLA (**A**), MLD (**B**), percent area stenosis (**C**) and lesion length (**D**) is shown. Abbreviations as in previous figures. * is represented multiplication sign.

Given these associations between stenosis parameters on the one hand and extent and intensity of ischemia on the other hand, we calculated the product of ischemic burden and relative signal intensity of ischemic areas as a surrogate parameter assessing at the same time both extent and severity of ischemia. As shown in Figure 5, this parameter significantly correlated with percent area stenosis (r = 0.435, *p* = 0.002), MLA (r = 0.382, *p* = 0.007), MLD (r = 0.417, *p* = 0.003) and lesion length (r = 0.295, *p* = 0.041).

Next, we analyzed whether plaque morphology is associated with ischemia in CMR. We found no significant association between total ischemic area and minimal fibrous cap thickness (r = 0.19, *p* = 0.332), mean fibrous cap thickness (r = 0.21, *p* = 0.292), lipid volume index (r = 0.30, *p* = 0.128) or extent of macrophage infiltration (macrophage arc: r = 0.15, *p* = 0.490).

### 3.3. Lesion-Derived Predictors of Clinically Relevant Ischemia

Current guidelines regarding CCS set the threshold for clinically relevant ischemia requiring revascularization at ≥10% of the myocardium [33,34]. In order to assess the performance of the various geometric and plaque-morphological features of the considered lesions, we performed ROC analysis for the prediction of an ischemic area >10% of the myocardium. Percent area stenosis (AUC 0.718, optimal cut-off 74.4%, sensitivity 84.6%, specificity 54.5%), MLA (AUC 0.732, optimal cut-off 1.13 mm², sensitivity 69.2%, specificity 77.3%), MLD (AUC 0.718, optimal cut-off 1.07 mm, sensitivity 73.1%, specificity 72.7%) and stenosis length (AUC 0.718, optimal cut-off 3.5 mm, sensitivity 92.3%, specificity 50.0%) predicted clinically relevant ischemia with good diagnostic efficiency.

**Figure 6:**
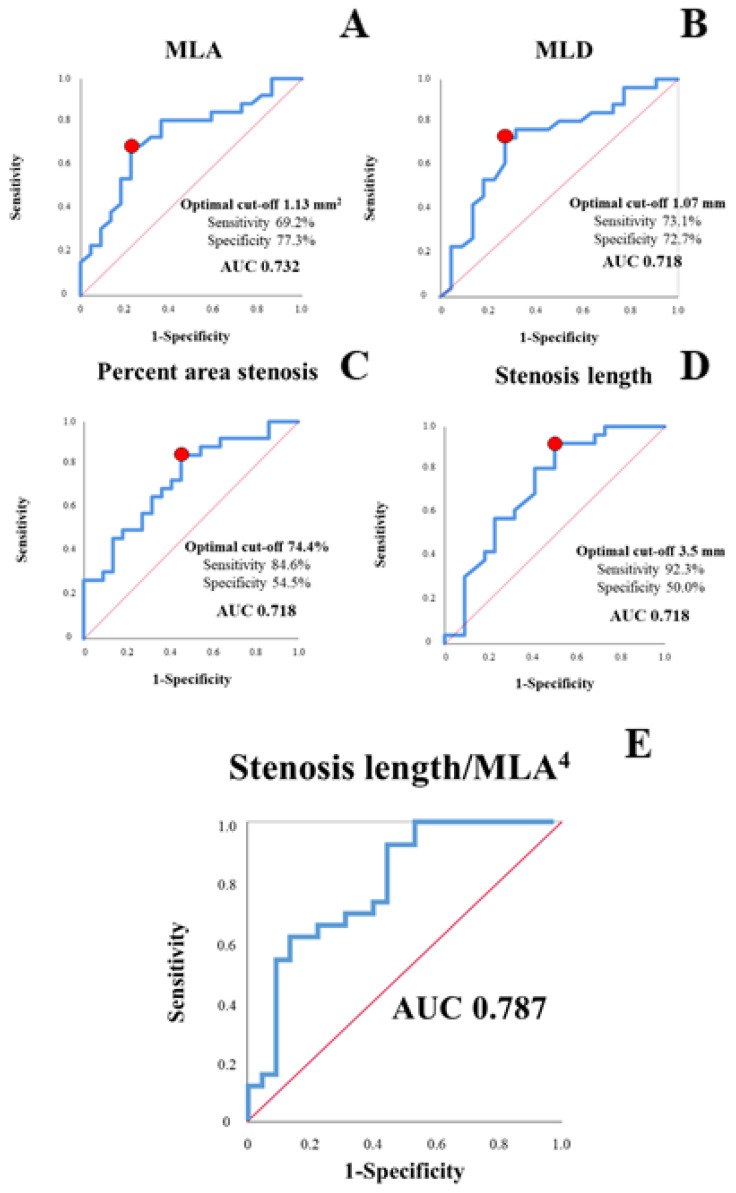
Analysis of the diagnostic efficiency of stenosis parameters alone (**A**–**D**) or in combination (**E**) in assessing ischemic area > 10% of total myocardium. Optimal cut-off is marked with a red circle. Abbreviations as in previous figures.

After demonstrating a good efficiency of single OCT-derived stenosis parameters in predicting clinically relevant ischemia, we tested whether a combination of these parameters could further improve their diagnostic efficiency. In multivariable regression analysis, various parameters assessing the severity of a stenosis (percent area stenosis, MLA, MLD) on the one hand and stenosis length on the other hand consistently showed an independent prediction of an ischemic burden >10%. After showing an independent prediction of clinically relevant ischemia, we tried to combine these parameters in order to test their diagnostic efficiency. We found that a Poiseuille-law based combination of stenosis length and MLA (stenosis length/MLA^4^) leads to a numeric increase in the AUC to 0.787 (Figure 6).

## 4. Discussion

The principal findings of the present study in patients with CCS are:

1. Intraluminal stenosis parameters assessing lesion severity are associated with the extent of myocardial ischemia as determined by CMR.
2. Plaque composition is not significantly related to extent of ischemia as determined by CMR.
3. OCT-derived stenosis parameters, alone and in combination, predicted ischemic area >10% of the myocardium with good diagnostic efficiency.

Several techniques are currently available to cardiologists to assess the hemodynamic relevance of coronary lesions and, consequently, to evaluate the need for coronary intervention. Among non-invasive methods, CMR has demonstrated its efficacy and cost-effectiveness [5,6,7]. Besides, CMR can also evaluate the extent of ischemia, which yields a relevant value in risk stratification [1,8]. The correlation between the presence of ischemia and geometry of a coronary lesion has already been shown [9,10,11,12,13,14,15,16,17]; however, to date, no study evaluated whether stenosis parameters predict also the extent of this ischemia. This may be clinically relevant, especially if there is no previous quantification of the ischemic burden because it may give interventionalists insights into the extent of ischemia based on the geometric features of the coronary lesion and may be a valuable addition to functional testings such as FFR or iFR.

### 4.1. Stenosis Geometry as Determined by OCT Is Associated with CMR Derived Ischemia

First, our study shows a solid association between OCT-derived geometric parameters describing the severity of coronary stenoses (such as percent area stenosis, MLA, MLD and lesion length) and the extent of myocardial ischemia as measured using CMR. On the one hand, this is in line with the understanding that ischemia reflects a lesion’s resistance, which in turn is determined by its geometry including its narrowing and length according to Poiseuille’s law (ΔP = 8µLQ/πR4) [36]. On the other hand, this is extremely interesting, because it demonstrates that the burden of ischemic myocardium is mainly influenced by the geometric properties of the stenosed epicardial vessel; however, other factors such as microvascular dysfunction, collateral growth and transmural redistribution of blood flow may still play a role, which needs to be further assessed in dedicated studies. However, due to the retrospective nature of our study and as OCT was usually performed in proximal lesions and/or in a major epicardial vessel likely to be of clinical relevance for the patient, the relationship between lesion geometry and extent of ischemia can only be applied to this lesion subset.

In addition, we extend current knowledge by quantifying the severity of ischemia by the relative signal intensity of ischemic areas and testing its relationship with stenosis parameters. We found that the anatomic severity of coronary stenoses was also associated with ischemic severity. Our data show that this CMR method may allow us to identify myocardium with a more pronounced perfusion deficit. It is tempting to speculate that ischemic severity in addition to ischemic burden may have a prognostic relevance—however, this needs to be investigated in further studies.

In contrast, no relevant association could be detected between ischemia in CMR and features of plaque vulnerability. This suggests that the negative prognostic value yield by a pathologic CMR study does not derive from a high-risk plaque phenotype, which, if not sealed [18], may lead to a higher rate of adverse events [37], but rather from other elements such as the extent of ischemia, presence of scar tissue and ventricular function.

### 4.2. OCT-Only Based Prediction of Clinically Relevant Ischemia

The current guidelines for revascularization in chronic coronary artery disease set the threshold for coronary intervention at ≥10% of ischemic myocardium. This threshold was supported by further studies showing an association between 10% ischemic myocardium on stress nuclear imaging and myocardial infarction or mortality risk due to coronary artery disease [8]. In our study, we developed a model to predict clinically relevant ischemia based on OCT-derived parameters. The good efficiency of this model with an AUC of 0.787 for the combination of MLA and lesion length makes OCT-derived lesion parameters a promising tool. Furthermore, the fact that again the best predictor of an ischemic burden >10% was modeled on Poiseuille’s law shows that the pressure drop caused by the anatomic severity of a coronary stenosis is the main factor in determining not only the presence but also the extent of ischemia.

### 4.3. Limitations

Although this is the first study to assess the association between OCT-derived geometry of coronary stenosis and CMR-derived ischemia, the patient population is still relatively small and our findings need to be confirmed in larger studies, specifically with regard to exact cut-off values. Additionally, the prognostic effects of extent and severity of ischemia cannot be conclusively evaluated on the basis of our study, due to the small patient size and to the observational study design; this relevant point needs to be assessed in further studies. Moreover, due to the exclusion of patients with chronic or acute kidney disease because of ethical reasons, we cannot draw any conclusion on this specific subgroup. Furthermore, as this study focused solely on patients with CCS, we cannot draw any conclusion on patients with acute coronary syndromes, who may more frequently express features of plaque vulnerability. Finally, due to the retrospective nature of our study, we cannot exclude any selection bias due to the inclusion criteria of our study.

## 5. Conclusions

Stenosis geometry in the coronary target segment as determined by OCT is significantly associated with both the extent and severity of myocardial ischemia as assessed by CMR. A Poiseuille’s law-modeled combination of lesion length and MLA could predict relevant ischemia with good efficiency. This highlights the key role of the geometry of epicardial stenoses in determining the pressure drop in coronary flow, which in turn influences the extent of ischemia.

## Figures and Tables

**Table 1:** Patient characteristics. Abbreviations: BMI = Body Mass Index, CAD = Coronary artery disease, ACEi = angiotensin-converting enzyme inhibitor, ARB = Angiotensin II receptor blockers, ASA = acetylsalicylic acid.

	*n* = 51
Age (years)	70.7 ± 8.0
Male sex (*n*, %)	39 (70.9)
**CV Risk profile**	
Diabetes mellitus (*n*, %)	43 (78.2)
BMI (kg/m²)	29.8 ± 3.8
Hypertension (*n*, %)	52 (94.5)
Dyslipidemia (*n*, %)	35 (63.6)
Current smoking (*n*, %)	7 (12.7)
Pack Years (PY)	18.3 ± 22.6
Family history of CAD (*n*, %)	22 (40.0)
**Lab values**	
Cholesterol (mg/dL)	194.0 ± 43.5
LDLc (mg/dL)	121.2 ± 34.8
HDLc (mg/dL)	44.4 ± 10.1
Triglycerides (mg/dL)	178.6 ± 84.2
hsCRP (mg/dL)	9.9 ± 11.6
HbA1c (%)	6.7 ± 1.0
**Medications**	
ACEi/ARB (*n*,%)	42 (77.8)
ß-Blocker (*n*,%)	48 (87.3)
ASA (*n*,%)	51 (92.7)
Statin (*n*,%)	37 (68.5)
Metformin (*n*,%)	31 (60.8)
Insulin (*n*,%)	17 (33.3)
Incretin-based therapy (*n*,%)	8 (15.7)

**Table 2:** OCT-derived lesion characteristics. Abbreviations: MLA = minimal lumen area, MLD = minimal lumen diameter, TCFA = thin capped fibroatheroma, FCT = fibrous cap thickness, LVI = lipid volume index.

	*n* = 55
**OCT-derived stenosis parameters**	
MLA (mm²)	1.4 ± 0.6
MLD (mm)	1.1 ± 0.2
Percent area stenosis (%)	75.0 ± 9.4
Lesion length (mm)	13.7 ± 7.4
Reference area (mm^2^)	5.8 ± 1.7
**OCT-derived plaque composition**	
Calcified plaque (*n*, %)	37 (62.7)
Fibrous plaque (*n*, %)	49 (83.1)
Lipid plaque (*n*, %)	25 (42.4)
TCFA (*n*, %)	10 (16.9)
Minimal FCT (µm)	82.4 ± 28.2
Mean FCT (µm)	125.8 ± 29.5
Mean lipid arc (°)	139.0 ± 51.3
LVI (mm *°)	617.2 ± 458.0
Presence of macrophages (*n*, %)	23 (41.8)
Mean macrophage arc (°)	16.1 ± 26.2

* is represented multiplication sign.

## Data Availability

All data are available on reasonable request from the corresponding author.
